# Health economic impacts associated with the consumption of sugar-sweetened beverages in Brazil

**DOI:** 10.3389/fnut.2022.1088051

**Published:** 2022-12-19

**Authors:** Joice Silva Vieira Leal, Aline Siqueira Fogal, Adriana Lúcia Meireles, Letícia de Oliveira Cardoso, Ísis Eloah Machado, Mariana Carvalho de Menezes

**Affiliations:** ^1^Graduate Program in Health and Nutrition, Universidade Federal de Ouro Preto, Ouro Preto, Brazil; ^2^Department of Clinical and Social Nutrition, Universidade Federal de Ouro Preto, Ouro Preto, Brazil; ^3^Department of Epidemiology, Oswaldo Cruz Foundation, National School of Public Health Sérgio Arouca (ENSP), Quantitative Methods in Health, Rio de Janeiro, Brazil; ^4^Department of Family Medicine, Mental and Collective Health, Universidade Federal de Ouro Preto, Ouro Preto, Brazil

**Keywords:** cost of illness, nutritional epidemiology, sugar-sweetened beverage, chronic non-communicable diseases, burden of disease

## Abstract

**Introduction:**

The consumption of sugar-sweetened beverages (SSBs) is among the main risk factors for non-communicable diseases (NCDs). This study aimed to estimate the financial costs of hospitalizations and procedures of high and medium complexity for NCDs attributable to the consumption of SSBs in the Brazilian Unified Health System (SUS) in 2019.

**Methods:**

This ecological study used data from the Global Burden of Disease (GBD) 2019 and the Department of Informatics of the Unified Health System (DATASUS). The attributable costs were estimated from the population-attributable fraction (PAF) and the costs in the treatment of chronic diseases [type 2 diabetes mellitus and ischemic heart disease (IHD)], stratified by sex, age group, level of complexity of treatment, and federative units.

**Results:**

In 2019, in Brazil, US$ 14,116,240.55 were the costs of hospitalizations and procedures of high and medium complexity in the treatment of NCDs attributable to the consumption of SSBs. These values were higher in males (US$ 8,469,265.14) and the southeast and southern regions, mainly in the state of São Paulo. However, when evaluating these results at a rate per 10,000 inhabitants, it was observed that the states of Paraná, Tocantins, and Roraima had higher costs per 10,000 inhabitants. Regarding the age groups, higher costs were observed in the older age groups.

**Conclusion:**

This study revealed the high financial impact of the NCDs treatment attributed to the consumption of SSBs in Brazil and the variability among Brazilian macro-regions. The results demonstrate the urgency and need for the expansion of policies to reduce the consumption of SSBs in Brazil with strategies that consider regional particularities.

## Introduction

The accelerated growth of non-communicable diseases (NCDs) has highlighted modifiable risk factors, including dietary risks (1). The World Health Organization points out that consumption of sugar-sweetened beverages (SSB) is among the main risk factors for chronic diseases and recommends limiting consumption of this group of beverages, including soft drinks and artificial juices (2). The prevalence of SSB consumption globally is demonstrated in recent studies, according to the national representative survey in Saudi Arabia 71.2% of adults consumed SSBs weekly, while 35.5% consumed SSBs daily (3). Another study conducted in the same country explored weekly and daily SSB consumption rates in a multiethnic population of middle-aged men, and showed that most individuals (93.8%) were weekly SSB consumers and about one-third (32.6%) were daily SSB consumers (4). In a national survey of Australian adults, 55.9 and 19.3% of men consumed SSBs weekly and daily, respectively (5). A Norwegian study reported that the rate of SSBs intake among adults was 34% (6). Another study indicated that 63.9% of American adults drank SSBs daily. In addition, the daily consumption rates of regular soda, fruit juices, and energy/sports drinks were 21, 6.6, and 5.7%, respectively (7). The daily consumption rate of SSBs among British adults was 20.4% (8). In Brazil, According to the last National Health Survey, 9.2% of the adult population regularly consume soft drinks (at least 5 days a week), being a more frequent habit among men and the younger population (9). Recent estimates of the Surveillance System of Risk and Protection Factors for Chronic Diseases by Telephone Survey reveal that the frequency of consumption of soft drinks on five or more days of the week was 14%, being higher among men (17.2%) than women (11.3%), considering the sample in Brazilian capitals (10).

The association between the consumption of SSBs and the development of diabetes and cardiovascular risk may be related to the high energy density of these beverages and their role in increasing the body weight, with obesity or overweight being an intermediate factor in this relationship. The biological mechanisms between these risk factors and the outcomes are mainly related to the high sugar content of these beverages, reduced satiety, and lack of compensatory mechanisms in calorie intake at subsequent meals (11). In addition, this development may stem from metabolic effects related to the high glycemic load (GL) of these beverages, which consequently induces a rapid elevation in blood glucose and insulin levels (11, 12). Diets with high GL may promote insulin resistance (12), increase inflammatory biomarkers (13), and are associated with an increased risk of type 2 diabetes mellitus (DM-2) (14, 15) and coronary heart disease (16). Although the association between the consumption of SSBs and NCDs has been well reported in the scientific literature (17), few studies have estimated the impact of this risk factor on the costs of health services, which pay for the treatment of these diseases. A recent study (18) estimated that in Brazil, approximately US$ 890 million were used in hospitalizations, outpatient procedures, and medications in the treatment of hypertension, diabetes, and obesity in the Unified Health System [Sistema Único de Saúde (SUS)] (18). A large part of these costs could be avoided by reducing the risk factors related to these diseases. An analysis of the impact of voluntary reduction of sodium in industrialized food would lead to savings of US$ 220 million in 20 years by reducing the total expenditure on the treatment of cardiovascular diseases (19). Further study of the impact of risk factors on the costs of different levels of healthcare is needed to direct priorities for investment in prevention policies (18). This study aimed to estimate the direct financial costs of hospitalizations and procedures of high and medium complexity for NCDs attributable to the consumption of SSBs in 2019 at the national level and in Brazilian federative units. Thus, it will be possible to obtain the cost values that could be saved in the country if the consumption of SSBs is reduced.

## Materials and methods

### Study design and population

This descriptive ecological study used secondary public domain data obtained from the Global Burden of Disease (GBD) 2019 study conducted by the Institute for Health Metrics and Evaluation and the Department of Informatics of the Unified Health System (DATASUS). The study population included adult individuals of both sexes, in all the federative units of Brazil, aged over 25 years; the GBD makes available estimates of the burden of NCDs attributed to dietary factors from the age of 25 years because it considers that the health effects resulting from these risk factors appear after this age (20).

### Load attributable to high consumption of SSBs

In this study, we evaluated the consumption of SSBs as a risk factor, defined as any intake (in grams per day) of beverages with ≥50 kcal per 226.8 g, including carbonated beverages, soft drinks, energy drinks, and fruit juices, excluding 100% fruit and vegetable juices, this exposure is defined by the GBD based on published systematic reviews (21).

The GBD selects the 24-h recall as the gold standard method of measuring food intake to assess the mean intake at the population level to ensure the comparability of data. SSBs intake was measured in a continuous and non-categorical manner (21). To estimate the mean daily intake of each risk factor, the GBD uses the Gaussian method standardized for country, year, age, and sex (22).

Two outcomes attributed to the consumption of SSBs were described and considered in the present study: DM-2 (23) described according to the International Statistical Classification of Diseases and Health Problems, version 10 (ICD-10) using the following codes: E11–E11.1 and E11.3–E11.9; and ischemic heart diseases (IHDs), with the following ICD-10 codes: I20–I21.6, I21.9–I25.9, and Z82.4–Z82.49 (20, 24). The IHDs represent the following diseases: acute myocardial infarction, chronic stable angina, chronic IHD, and heart failure due to IHD, all of which were identified using standard case definitions. Myocardial infarction was defined according to the Fourth Universal Definition of Myocardial Infarction and adjusted to include out-of-hospital sudden cardiac death. Stable angina was defined using the Rose Angina Questionnaire (25).

The 2019 GBD, from reviews in the scientific literature, considers two metabolic risk factors as mediators for the outcome of DM-2: elevated fasting plasma glucose and body mass index (BMI), which are described as factors involved in the causal pathway of DM-2 development related to SSBs consumption (21).

To estimate the fraction of the attributable burden or population-attributable fraction (PAF) of each outcome (DM-2 and IHD) to the consumption of SSBs, three components were used: (a) the level of risk factor exposure, that is, the average daily consumption of SSBs by the population, (b) the counterfactual level of risk factor exposure or theoretical minimum risk exposure level (TMREL), and (c) the relative risk of the outcome due to exposure (consumption of SSBs) relative to the TMREL (23).

To determine the mean TMREL value, the GBD conducted a systematic review of the scientific literature to identify national or subnational representative surveys that provide data on the dietary intake of SSBs in countries (22). The TMREL value for some harmful dietary factors with uniform risk function growth was set to 0 g, as is the case with the consumption of SSBs (21). The relative risks in relation to the TMREL were obtained from surveys in published systematic reviews. PAF represents the proportion of NCDs that would be reduced or eliminated in Brazil in 2019 if exposure to a particular risk factor (such as consumption of SSBs) in the past was reduced to an ideal exposure scenario (0 g/day) (21, 26). The PAF value is age-, sex-, location-, and year-specific, allowing for stratified analyses (27).

The PAF values for each outcome attributed to the consumption of SSBs (DM-2 and DIC) were extracted from the GBD. Since obesity is a mediating factor in the causal pathway of SSBs consumption and DM-2 (21), GBD does not estimate the PAF of obesity. However, since obesity is described as a diagnosis in the SUS databases, to obtain the PAF of obesity attributed to the consumption of SSBs, we chose, by convention, to use the same PAF as for DM-2.

### Estimated financial costs

We estimated the direct costs allocated to the treatment of NCDs attributed to the consumption of SSBs, which refer to direct care to the individual and include hospital admissions, outpatient procedures, examinations, tests and controls, inputs, medications, emergency services, employee compensation, nursing services, and other services directly linked to patient care (27, 28).

To estimate the financial costs, the hospital information system (SIH) and outpatient information system (SIA), available *via* DATASUS, were used as data sources. These databases were used because they provide the cost per procedure related to the main disease available from the ICD-10 code (“DATASUS”, [S.d.]). Thus, the variable “ICD-10 codes” was used to link the information from SIH and SIA to the PAF made available by the GBD.

The database was extracted from the SIA data files [individualized outpatient production bulletin–BPA I and authorization of high complexity procedures (APAC) and SIH (authorization of hospital admissions, AIH)] on the DATASUS website for the years 2019, 2020, and 2021. Subsequently, only procedures and hospitalizations performed in 2019 were included. For the extraction and processing of these data, the microdatasus package was used in the statistical program R (29).

After the data extraction and processing step in the R program, the ICD-10 package of the STATA software, version 15, was used to group the outcomes that generated the procedures, from the ICD-10 codes, into the GBD 2019 causes attributable to the consumption of SSBs (DM-2 and DIC).

Finally, the Microsoft Excel 2019 program was used to combine the information on PAF values for the year 2019, obtained from the GBD study, with information on total costs by outpatient procedures and hospitalizations, by age group, sex, and federative units.

After grouping the data, the financial costs per NCDs attributable to the consumption of SSBs were obtained by multiplying the total values of hospitalizations and high and medium complexity procedures by the corresponding PAF for each location (Brazil and federative units), sex, and age group. In addition, for comparison purposes among the federative units, the total values per federative unit were divided by the resident population according to data provided by the DATASUS and multiplied by 10,000 inhabitants.

These costs were obtained in reais (Brazilian currency) and converted into US$–dollars, considering the 2019 exchange rate according to the Organization for Economic Cooperation and Development (OECD) (US$ 3.944). The cost values in the treatment of NCDs attributed to the regular consumption of SSBs were presented by age group, sex, outcome, federative unit, Brazilian macro-region, and level of complexity (outpatient or inpatient).

## Results

In Brazil, the costs in 2019 were US$ 14,116,240.55 (equivalent to R$ 55,674,452.74), with high and medium complexity procedures in the treatment of NCDs attributed to the consumption of SSBs. These values were higher in males (US$ 8,469,265.14) than in females (US$ 5,646,975.42). The costs of NCDs treatment attributed to the consumption of SSBs were US$ 162,277.61 for DM-2, US$ 1,572,456.50 for obesity, and US$ 12,381,506.46 for IHD (Table 1).

**TABLE 1:** Financial costs in the treatment of NCDs attributed in the consumption of SSBs, international dollars (US$), in the year 2019, by sex, age group, regions and Brazilian federative units, and outpatient information system (SIA) and hospital information system (SIH), according to Global Burden of Disease (GBD) 2019.

Age (years)										
Location	Sex	25 to 29	30 to 34	35 to 39	40 to 44	45 to 49	50 to 54	55 to 59	60 or more	Total
		SIA e SIH	SIA e SIH	SIA e SIH	SIA e SIH	SIA e SIH	SIA e SIH	SIA e SIH	SIA e SIH	
Brazil	Female	241,359.31	316,177.63	351,747.27	399,193.06	465,976.94	600,252.92	699,046.69	2,573,221.60	**5,646,975.42**
	Male	80,883.84	132,740.77	240,060.23	356,684.31	650,253.17	1,030,197.56	1,331,155.07	4,647,290.18	**8,469,265.14**
	**Total**	**322,243.15**	**448,918.41**	**591,807.50**	**755,877.37**	**1,116,230.11**	**1,630,450.48**	**2,030,201.76**	**7,220,511.78**	**14,116,240.55**
North	**810,216.43**									
Acre	Female	271.54	385.51	138.00	217.72	188.29	378.65	775.63	2,705.70	
	Male	66.29	482.29	101.58	593.42	915.96	1,064.03	1,638.93	5,431.73	
	**Total**	**337.83**	**867.81**	**239.58**	**811.14**	**1,104.25**	**1,442.69**	**2,414.56**	**8,137.43**	**15,355.27**
Amapá	Female	–	368.80	86.52	212.35	796.11	1,055.18	1,348.77	5,386.70	
	Male	73.43	119.10	96.94	797.80	2,243.88	2,219.23	3,255.80	12,268.22	
	**Total**	**73.43**	**487.90**	**183.46**	**1,010.15**	**3,039.99**	**3,274.41**	**4,604.56**	**17,654.92**	**30,328.83**
Amazonas	Female	304.23	174.11	395.70	2,048.64	2,656.13	2,387.11	5,087.94	17,048.43	
	Male	217.62	1,249.10	2,020.50	3,665.21	7,523.15	10,955.51	12,122.25	44,350.01	
	**Total**	**521.85**	**1,423.20**	**2,416.20**	**5,713.85**	**10,179.28**	**13,342.62**	**17,210.19**	**61,398.44**	**112,205.63**
Pará	Female	326.91	805.37	809.35	1,835.60	3,071.55	4,316.22	5,403.24	21,748.53	
	Male	171.61	705.61	1,588.75	2,831.47	5,144.22	11,954.24	14,647.27	57,460.54	
	**Total**	**498.51**	**1,510.97**	**2,398.10**	**4,667.07**	**8,215.77**	**16,270.46**	**20,050.51**	**79,209.07**	**132,820.46**
Rondônia	Female	136.54	1,609.07	186.03	600.97	841.58	1,990.25	1,222.48	6,473.80	
	Male	282.72	219.79	2,240.03	635.84	3,202.91	4,135.89	5,991.42	21,757.25	
	**Total**	**419.26**	**1,828.86**	**2,426.06**	**1,236.81**	**4,044.50**	**6,126.15**	**7,213.90**	**28,231.06**	**51,526.59**
Roraima	Female	741.09	99.19	216.71	775.40	224.03	380.58	722.29	1,659.82	
	Male	3.13	7.00	358.24	448.56	999.65	1,555.32	2,424.71	5,074.78	
	**Total**	**744.22**	**106.19**	**574.96**	**1,223.97**	**1,223.68**	**1,935.90**	**3,147.00**	**6,734.60**	**15,690.52**
Tocantins	Female	643.65	568.54	675.45	1,941.48	1,183.56	1,911.66	1,937.45	8,931.70	
	Male	297.22	178.85	282.79	1,846.60	2,361.85	3,320.28	5,247.08	21,106.89	
	**Total**	**940.87**	**747.39**	**958.24**	**3,788.08**	**3,545.41**	**5,231.94**	**7,184.53**	**30,038.59**	**52,435.05**
Northeast	**2,270,719.50**									
Alagoas	Female	930.54	506.61	1,906.95	2,404.47	3,713.16	6,052.01	6,856.72	23,911.23	
	Male	114.47	579.52	996.12	2,900.68	6,110.81	8,059.23	10,755.14	41,262.84	
	**Total**	**1,045.01**	**1,086.14**	**2,903.07**	**5,305.15**	**9,823.97**	**14,111.24**	**17,611.86**	**65,174.07**	**117,060.51**
Bahia	Female	1,564.17	3,136.48	3,752.98	7,765.65	13,139.48	24,372.88	29,242.80	123,913.72	
	Male	1,688.90	3,217.03	6,482.95	12,724.77	22,382.03	39,580.39	51,610.01	188,221.14	
	**Total**	**3,253.06**	**6,353.51**	**10,235.93**	**20,490.41**	**35,521.51**	**63,953.26**	**80,852.81**	**312,134.87**	**532,795.36**
Ceará	Female	1,635.32	3,002.61	3,678.38	6,086.84	10,155.70	11,143.99	18,981.21	101,279.80	
	Male	1,530.74	2,623.47	5,076.88	11,056.26	16,965.90	32,549.08	45,311.52	180,817.17	
	**Total**	**3,166.06**	**5,626.08**	**8,755.26**	**17,143.10**	**27,121.60**	**43,693.07**	**64,292.73**	**282,096.97**	**451,894.88**
Maranhão	Female	334.80	739.29	1,558.63	1,715.19	2,402.58	2,971.48	4,917.82	22,631.56	
	Male	251.95	1,192.87	994.61	2,198.50	4,655.96	6,363.60	12,010.42	43,911.55	
	**Total**	**586.75**	**1,932.16**	**2,553.25**	**3,913.69**	**7,058.53**	**9,335.08**	**16,928.25**	**66,543.11**	**108,850.80**
Paraíba	Female	1,169.44	1,544.54	1,844.55	2,248.78	4,600.57	5,636.53	9,739.23	43,556.54	
	Male	345.14	660.23	2,164.54	4,758.91	7,384.71	11,214.41	15,724.67	68,507.27	
	**Total**	**1,514.58**	**2,204.77**	**4,009.08**	**7,007.69**	**11,985.28**	**16,850.94**	**25,463.90**	**112,063.81**	**181,100.04**
Pernambuco	Female	3,935.29	6,573.54	7,258.66	15,195.23	11,634.79	18,302.52	24,509.97	108,085.63	
	Male	1,437.73	2,831.27	5,126.15	10,520.67	22,083.98	31,384.13	41,658.30	162,562.88	
	**Total**	**5,373.02**	**9,404.81**	**12,384.81**	**25,715.90**	**33,718.78**	**49,686.65**	**66,168.27**	**270,648.51**	**473,100.75**
Piauí	Female	51.23	159.02	597.12	818.24	1,546.89	1,847.56	3,500.00	19,932.19	
	Male	85,25	302,49	1.066,03	2.603,55	3.243,62	7.115,18	8,664.79	37,222.13	
	**Total**	**136.49**	**461.51**	**1,663.15**	**3,421.79**	**4,790.50**	**8,962.73**	**12,164.80**	**57,154.33**	**88,755.31**
Rio Grande do Norte	Female	606.65	2,455.39	2,617.01	2,741.61	4,907.86	8,908.47	11,472.30	49,354.77	
	Male	1,184.50	1,768.87	3,803.70	5,972.97	11,252.89	17,327.47	27,201.20	79,554.01	
	**Total**	**1,791.15**	**4,224.26**	**6,420.70**	**8,714.58**	**16,160.75**	**26,235.94**	**38,673.51**	**128,908.78**	**231,129.68**
Sergipe	Female	231.28	184.43	363.13	2,596.80	2,643.43	2,731.31	4,374.58	17,665.19	
	Male	189.19	407.60	2,652.94	2,261.51	4,396.84	6,682.53	8,125.23	30,526.17	
	**Total**	**420.47**	**592.03**	**3,016.06**	**4,858.31**	**7,040.27**	**9,413.84**	**12,499.80**	**48,191.37**	**86,032.16**
Southeast	**5,891,367.54**									
Espírito Santo	Female	7,625.32	10,895.69	15,587.91	16,408.80	14,501.39	19,738.70	18,982.95	75,447.47	
	Male	1,473.65	3,370.15	4,767.28	7,654.08	14,927.73	22,379.80	32,656.78	119,142.55	
	**Total**	**9,098.97**	**14,265.84**	**20,355.18**	**24,062.88**	**29,429.12**	**42,118.51**	**51,639.72**	**194,590.02**	**385,560.24**
Minas Gerais	Female	12,077.62	23,907.62	28,182.57	41,388.96	53,608.38	68,499.99	77,045.12	303,756.16	
	Male	5,505.60	12,949.14	23,633.42	36,841.74	74,775.45	119,002.32	155,567.98	584,279.21	
	**Total**	**17,583.22**	**36,856.76**	**51,815.99**	**78,230.70**	**128,383.83**	**187,502.31**	**232,613.09**	**888,035.37**	**1,621,021.28**
Rio de Janeiro	Female	2,579.66	3,174.83	7,066.55	9,075.36	22,377.96	32,060.83	44,430.72	161,260.38	
	Male	2,514.97	4,965.41	12,175.39	20,507.95	36,646.11	66,710.21	86,070.31	277,882.48	
	**Total**	**5,094.64**	**8,140.24**	**19,241.94**	**29,583.31**	**59,024.07**	**98,771.04**	**130,501.03**	**439,142.87**	**789,499.14**
São Paulo	Female	30,414.74	51,548.59	68,270.78	77,534.49	102,595.73	145,751.86	165,696.17	567,785.13	
	Male	18,633.04	31,613.52	64,248.86	93,252.56	165,552.04	253,711.02	311,092.70	947,585.66	
	**Total**	**49,047.78**	**83,162.11**	**132,519.64**	**170,787.05**	**268,147.76**	**399,462.88**	**476,788.87**	**1,515,370.79**	**3,095,286.87**
Midwest	**934,055.85**									
Distrito Federal	Female	476.48	733.37	2,271.90	3,269.99	6,820.68	8,728.10	8,364.74	31,358.08	
	Male	332.66	1,607.94	3,996.10	4,908.43	15,865.91	14,271.01	17,509.78	53,322.94	
	**Total**	**809.14**	**2,341.31**	**6,268.00**	**8,178.42**	**22,686.59**	**22,999.11**	**25,874.53**	**84,681.02**	**173,838.10**
Goiás	Female	6,021.15	7,852.15	9,367.08	12,797.20	13,714.75	15,631.25	21,454.97	74,846.81	
	Male	2,425.99	3,997.89	7,403.59	9,154.15	15,346.75	26,602.90	34,703.15	121,669.48	
	**Total**	**8,44714**	**11,850.03**	**16,770.68**	**21,951.35**	**29,061.49**	**42,234.15**	**56,158.12**	**196,516.28**	**382,989.24**
Mato Grosso	Female	190.50	968.40	2,503.39	2,989.96	3,440.13	6,867.65	8,968.49	29,947.95	
	Male	675.10	2,009.39	2,703.87	4,627.77	7,856.31	15,211.25	15,187.88	50,273.47	
	**Total**	**865.60**	**2,977.79**	**5,207.25**	**7,617.72**	**11,296.44**	**22,078.90**	**24,156.37**	**80,221.42**	**154,421.49**
Mato Grosso do Sul	Female	122.59	1,184.95	996.80	3,496.28	4,212.18	7,233.66	10,870.45	41,482.26	
	Male	679.05	1,447.04	2,238.08	5,070.00	12,148.31	18,269.49	22,942.78	90,413.07	
	**Total**	**801.65**	**2,631.99**	**3,234.89**	**8,566.28**	**16,360.49**	**25,503.16**	**33,813.24**	**131,895.33**	**222,807.02**
**South**	**4,609,735.29**									
Paraná	Female	159,101.32	171,529.63	158,217.06	137,578.49	123,372.52	120,979.07	118,956.47	368,005.29	
	Male	34,229.38	38,767.44	52,785.26	62,168.96	89,729.27	136,609.57	179,094.71	719,207.46	
	**Total**	**193,330.70**	**210,297.07**	**211,002.32**	**199,747.45**	**213,101.78**	**257,588.64**	**298,051.18**	**1,087,212.75**	**2,670,331.89**
Rio Grande do Sul	Female	4,561.64	11,815.74	18,221.17	25,512.65	33,949.72	43,632.73	53,388.43	214,415.85	
	Male	4,090.90	8,271.81	15,648.16	24,667.14	53,546.89	90,592.54	116,999.55	411,437.43	
	**Total**	**8,652.53**	**20,087.55**	**33,869.33**	**50,179.79**	**87,496.60**	**134,225.27**	**170,387.98**	**625,853.28**	**1,130,752.34**
Santa Catarina	Female	5,305.60	10,254.16	14,976.90	19,935.90	23,677.81	36,742.67	40,795.74	130,630.90	
	Male	2,383.62	7,195.96	15,407.47	22,014.81	42,990.04	71,356.92	92,940.71	272,041.84	
	**Total**	**7,689.22**	**17,450.12**	**30,384.37**	**41,950.72**	**66,667.85**	**108,099.59**	**133,736.46**	**402,672.74**	**808,651.06**

SIA, outpatient information system; SIH, hospital information system. Bold values represent the international dollars (US$).

Regarding age groups, higher costs were observed in the older age groups, and an increase in SUS costs with high and medium complexity procedures in the treatment of NCDs attributable to the consumption of SSBs, corresponding to a total cost with individuals over 60 years old of US$ 7,220,511.78.

**FIGURE 1:**
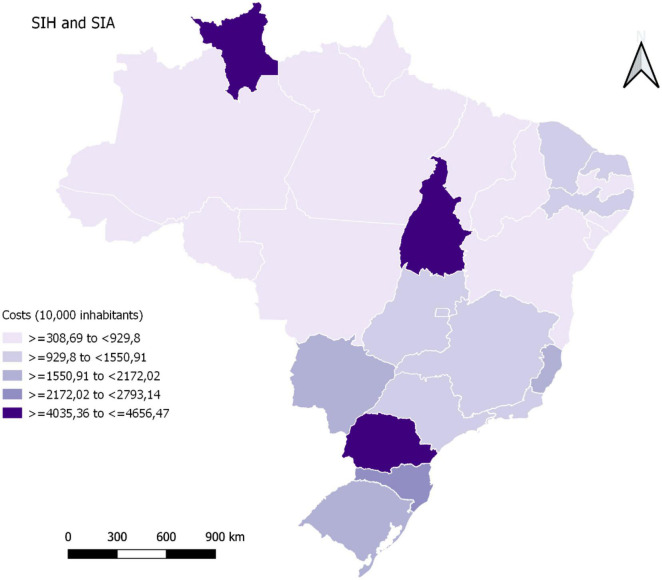
Financial costs in the treatment of non-transmissible chronic diseases (NCDs) attributed to the consumption of sugar-sweetened beverages (SSBs), in international dollars (US$) and in the federative units per 10,000 inhabitants, Brazil, 2019.

When evaluated according to the state, the states of São Paulo (US$ 3,095,286.87) and Paraná (US$ 2,670,331.89) had the highest total costs, and the states of Acre (US$ 15,355.27) and Roraima (US$ 15,690.52) had the lowest financial costs in the treatment of NCDs attributed to the consumption of SSBs (Table 1). For the Brazilian macro-regions, the southeast and southern regions presented the highest cost values, as shown in Figure 1.

When evaluating these costs per 10,000 inhabitants, it was observed that the states of Paraná, Tocantins, and Roraima obtained the highest costs in the treatment of NCDs attributed to the consumption of SSBs in high and medium complexity procedures in the SUS in 2019 (Figure 1).

Table 2 shows the financial costs of the SUS in procedures (inpatient and outpatient) according to the SIH and SIA by sex, age group, and outcome. The results reveal that the cost values in 2019 with inpatient procedures were higher than those with outpatient procedures, regardless of the outcome, age group, and sex.

**TABLE 2:** Financial costs of hospitalizations and high and medium complexity procedures for NCDs attributed to the consumption of SSBs, in international dollars (US$), by age group, sex, and outcomes, Brazil, 2019, SIA and SIH, according to GBD 2019.

Outcome	Age group	Female						Male						Total
		SIA	II 95%		SIH	II 95%		SIA	II 95%		SIH	II 95%		
Type 2 diabetes mellitus	**25 to 29**	95.21	28.03	217.35	2,939.59	862.85	6,841.95	55.99	19.47	125.93	3,005.58	1,011.76	6,746.82	
	**30 to 34**	128.94	38.89	293.65	5,031.69	1,511.21	11,813.31	189.74	63.32	429.01	3,256.81	1,117.60	7,373.08	
	**35 to 39**	228.95	71.40	539.02	4,446.86	1,318.18	10,594.06	208.51	81.91	471.01	6,446.69	2,479.63	14,824.94	
	**40 to 44**	306.85	101.96	716.05	5,533.03	1,784.49	13,080.88	359.52	146.16	800.50	4,942.66	1,957.93	10,970.99	
	**45 to 49**	503.29	171.04	1,207.51	6,037.77	1,976.24	14,601.28	587.55	250.14	1,279.70	8,394.99	3,509.01	18,831.67	
	**50 to 54**	641.56	218.22	1,556.77	8,708.63	2,964.10	21,015.50	682.01	290.06	1,444.62	11,657.58	4,813.51	26,424.11	
	**55 to 59**	718.51	261.61	1,673.92	8,659.70	3,193.42	20,237.81	714.41	321.28	1,450.95	11,097.84	4,696.86	23,848.61	
	**60 to 64**	837.89	301.83	1,904.39	8,552.63	2,915.81	20,284.46	742.40	303.64	1,462.77	9,768.05	3,976.20	20,239.42	
	**65 to 69**	548.97	200.90	1,266.29	8,008.01	2,797.94	18,531.62	506.70	234.21	966.07	8,653.65	3,821.92	17,326.63	
	**70 to 74**	362.39	134.10	811.29	6,354.63	2,281.41	14,560.75	294.89	125.59	585.70	6,531.56	2,692.93	13,354.98	
	**75 to 79**	156.06	55.50	346.73	4,370.72	1,554.00	10,091.63	196.28	83.92	368.68	3,461.64	1,480.86	6,760.23	
	**80 to 84**	69.66	25.13	140.29	2,095.62	787.55	4,312.63	52.45	23.48	93.13	1,871.42	824.57	3,475.39	
	**85 to 89**	21.58	5.45	62.35	850.42	207.52	2,477.20	9.30	2.67	24.50	420.37	118.40	1,109.72	
	**90 to 94**	5.34	1.28	15.76	160.60	40.61	473.75	5.81	1.58	16.04	28.67	8.10	80.71	
	**95 or more**	20.59	4.58	60.33	895.50	210.41	2,673.93	35.50	9.64	103.22	807.85	232.24	2,276.76	
	**Total**	**4,645.79**	1,619.92	10,811.70	**72,645.40**	24,405.74	171,590.76	**4,641.06**	1,957.07	9,621.83	**80,345.36**	32,741.52	173,644.06	**162,277.61**
Ischemic heart disease	**25 to 29**	2,917.99	381.45	7,087.60	16,767.57	2,134.47	42,297.10	3,458.19	552.03	8,361.11	36,895.90	5,848.10	89,998.67	
	**30 to 34**	5,366.20	691.72	13,031.97	37,405.56	4,829.75	92,934.37	5,985.48	990.90	14,699.28	75,706.99	12,528.43	187,046.06	
	**35 to 39**	11,991.87	1,622.93	30,278.12	77,995.85	10,655.37	198,826.07	12,680.38	2,316.80	31,286.12	172,924.85	31,340.54	431,289.80	
	**40 to 44**	21,460.71	3,481.43	54,701.78	153,111.00	24,327.52	396,149.88	20,915.54	4,267.55	49,804.35	292,620.86	60,776.54	706,498.49	
	**45 to 49**	37,815.41	6,584.60	95,745.25	278,031.77	49,108.68	716,775.61	37,338.70	8,294.40	85,027.85	576,885.53	129,237.90	1,328,393.68	
	**50 to 54**	57,995.10	10,418.40	144,960.28	418,121.92	75,968.66	1,054,518.47	60,483.80	13,816.04	135,159.69	939,658.51	219,442.67	2,092,787.98	
	**55 to 59**	66,650.45	14,625.18	156,501.35	550,330.14	120,479.58	1,298,783.04	77,372.64	19,465.85	160,545.69	1,231,048.70	312,118.83	2,546,843.67	
	**60 to 64**	71,448.68	16,175.84	161,438.64	616,910.04	140,419.75	1,396,146.83	85,502.52	22,313.81	168,318.88	1,373,289.48	357,068.57	2,700,899.10	
	**65 to 69**	63,193.99	15,082.68	139,341.32	590,446.66	141,060.11	1,295,283.11	76,855.59	20,549.53	147,681.75	1,212,163.18	327,733.03	2,324,708.55	
	**70 to 74**	46,032.41	11,437.82	100,207.31	475,237.94	117,786.92	1,040,717.08	54,041.05	14,814.15	108,136.72	848,597.63	230,805.55	1,686,698.27	
	**75 to 79**	28,234.05	7,199.46	62,698.66	321,772.48	81,422.02	721,749.79	33,091.33	9,094.38	66,767.81	541,999.59	149,910.79	1,088,784.56	
	**80 to 84**	13,114.02	3,367.80	27,705.76	178,267.64	44,939.22	379,377.89	14,941.04	3,959.91	30,070.00	262,415.40	69,608.61	519,558.33	
	**85 to 89**	3,385.39	725.18	9,217.35	65,122.73	13,942.83	176,793.35	3,622.24	789.88	9,132.09	78,570.27	17,174.80	195,994.22	
	**90 to 94**	698.37	147.29	1,953.18	18,919.76	4,016.25	52,444.79	601.24	130.62	1,554.16	17,095.87	3,716.66	43,522.39	
	**95 or more**	122.37	26.34	340.31	3,304.19	705.51	9,344.97	131.31	28.18	340.99	2,440.39	532.08	6,230.91	
	**Total**	**430,427.01**	91,968.12	1,005,208.88	**3,801,745.25**	831,796.64	8,872,142.35	**487,021.05**	121,384.03	1,016,886.49	**7,662,313.15**	1,927,843.10	15,949,254.68	**12,381,506.46**
Hight BMI (PAF DM2)	**25 to 29**	2,212.27	644.08	4,950.87	216,426.67	62,856.52	481,189.18	486.03	160.58	1,085.29	36,982.16	12,194.58	83,015.35	
	**30 to 34**	3,082.61	849.82	7,049.96	265,162.64	72,760.21	602,099.74	472.80	163.74	1,015.31	47,128.95	16,182.88	101,220.79	
	**35 to 39**	2,857.04	887.05	6,618.95	254,226.71	77,739.65	586,670.78	542.54	209.36	1,178.33	47,257.27	18,140.86	103,248.87	
	**40 to 44**	2,333.19	701.39	5,521.66	216,448.28	64,706.25	512,806.24	456.78	189.37	1,023.50	37,388.95	15,425.79	84,176.57	
	**45 to 49**	1,771.44	595.65	4,219.69	141,817.25	46,888.73	337,009.86	333.97	137.05	755.73	26,712.44	10,876.48	61,146.58	
	**50 to 54**	1,508.80	506.63	3,675.64	113,276.91	37,626.05	277,077.00	214.30	87.09	488.90	17,501.37	7,073.02	40,302.37	
	**55 to 59**	1,007.81	353.58	2,492.68	71,680.07	24,801.01	180,797.18	203.80	87.86	438.95	10,717.68	4,675.85	23,156.29	
	**60 to 64**	653.85	227.45	1,527.31	29,328.08	9,838.52	70,593.83	118.54	52.48	239.39	6,047.67	2,686.01	12,437.28	
	**65 to 69**	292.85	100.03	690.50	9,966.91	3,537.06	24,223.07	67.30	30.92	130.66	1,964.47	895.28	4,014.95	
	**70 to 74**	128.69	45.20	306.69	1,960.38	703.36	4,667.98	29.85	12.90	56.40	240.46	108.65	486.62	
	**75 to 79**	37.42	13.10	88.31	206.68	72.68	526.48	9.98	4.39	19.37	–	–	–	
	**80 to 84**	13.48	4.90	28.73	456.95	186.59	1,066.86	1.52	0.66	2.78	62.61	26.44	126.50	
	**85 to 89**	7.62	1.83	23.42	491.00	122.18	1,515.57	1.48	0.41	3.87	–	–	–	
	**90 to 94**	10.17	2.84	32.81	142.46	34.33	440.10	1.64	0.49	4.04	–	–	–	
	**95 or more**	3.71	1.03	11.01	–	–	–	–	–	–	–	–	–	
	**Total**	**15,920.95**	4,934.58	37,238.23	**1,321,590.99**	401,873.14	3,080,683.87	**2,940.53**	1,137.30	6,442.52	**232,004.03**	88,285.84	513,332.17	**1,572,456.50**

SIA, outpatient information system; SIH, hospital information system; BMI, body mass index; PAF, population attributable fraction; DM-2, type 2 diabetes mellitus. Bold values represent the international dollars (US$).

## Discussion

In 2019, $ 14,116,240.55 was used in the medium and high complexity procedures in the treatment of NCDs attributed to the consumption of SSBs, in the SUS. These values were higher in males and showed geographic variability.

According to data from the Health Economics Bulletin in 2019 from the Brazilian Ministry of Health (2021), the Brazilian government invested approximately 4% of the Gross Domestic Product in public health actions and services, totaling R$ 289.6 billion (30). According to information made available by the Pan American Health Organization, from 2010 to 2015, there was a global increase in average costs of public health services from 3.8 to 4.2% (1, 2). NCDs are a global health problem that directly impacts the costs of countries’ health systems, and their main risk factors are behavioral and modifiable, such as the consumption of SSBs. Analyzing the healthcare costs of other risk factors for NCDs, Ding et al. (31) report that the global estimate of costs related to physical inactivity was $ 53.8 billion in 2013. Therefore, several strategies have been adopted globally to reduce the health impact of NCDs and their risk factors (31).

Regarding the risk factor studied, in recent years, several countries have adopted policies with the intention of reducing the consumption of SSBs in the population, including taxing them at higher rates. In some places, these measures have proven effective, as is the case in Mexico (32). A cross-sectional study conducted in different countries demonstrated the relevance of tax policies by establishing a relationship between the affordability of SSBs and the prevalence of overweight and obesity (33). Age-standardized prevalence rates of overweight and obesity have increased due to the increased affordability of SSBs, reinforcing current recommendations that fiscal policies and other actions are important to encourage a reduction in the consumption of SSBs and control population weight gain (34).

However, in Brazil, this is still an agenda under discussion among researchers, civil society, and public powers (35, 36). Despite the great debate around policies to reduce the consumption of SSBs, there are currently observed actions contrary to these policies, such as the wide range of tax benefits granted to the SSB production industry, culminating in the reduction of the final price of their products, making them more accessible to the population (35).

The values of costs for NCDs treatment attributable to the consumption of SSBs were higher in males compared with females, this may be related to the higher consumption of SSBs by males, as revealed by the latest population surveys conducted in Brazil, such as the VIGITEL of 2021, which showed that the consumption of soft drinks in the Brazilian population is higher in men (17.2%) than that in women (11.3%) (10). In addition, the National Health Survey (PNS) 2019 pointed out that 9.2% of Brazilian adults regularly consumed soft drinks, being a more frequent habit among men (11.6%) than that of among women (7.2%) (9).

The values of costs for NCDs treatment attributable to the consumption of SSBs were also higher in the southeast region, especially in the state of São Paulo, followed by the states of the southern region; however, when evaluating these results in a rate per 10,000 inhabitants, it was observed that the states of Paraná, Tocantins, and Roraima, which belong to the northern region, had higher values of costs. These findings may be related to inequalities in access to healthcare in Brazil and regional diversity (37). Studies have shown that access to and use of health services in Brazil reflects inequalities among distinct social groups (38). The data found in the last PNS corroborate these studies, revealing that the Brazilian regions with the highest proportions seeking healthcare were the southeast (20.9%) and southern (19.8%) regions with better living conditions and higher human development indexes, while the northern region had the lowest proportion (13.7%) (9).

This study showed that the total values for outpatient procedures were lower than those for inpatient services in 2019. This is due to the higher amount paid in inpatient procedures, thus totaling a larger sum than that for outpatient services (39). It is noteworthy that the care model proposed by the SUS is a highly effective and efficient way to act on the main causes of health problems and risks to wellbeing, as well as deal with emerging challenges that threaten health and wellbeing in the future. Primary health care is a cost-effective investment, as there is evidence that quality primary care reduces total healthcare costs and improves efficiency, for example, by reducing hospital admissions. Thus, strengthening this model of care is important for addressing NCDs and their risk factors, as well as for being cost-effective. Furthermore, strengthening systems in the community with the decentralization of health services contribute to building resilience, which is fundamental to resisting shocks in health systems (1, 2).

The results of this study should be interpreted in light of its limitations. The diversity and plurality of the Brazilian regions can be interfering factors, for example, with the different quality of the databases, due to the difficulty of access to a quality service, because of availability and geographical distance, both in the northern and northeast regions, the estimates collected from these regions are limiting (38). Furthermore, caution is recommended when interpreting the results of the federative unit and sex because of the amplitude of the uncertainty intervals, which sometimes overlap.

In addition, data on the consumption of SSBs come from various sources, and the 2019 GBD does not yet include more recent sources that estimate SSBs consumption, such as the Household Budget Survey (POF) 2017–2018 and National Health Survey 2019. Finally, it is noteworthy that the cost estimates reflect only the data in the SUS and do not include cost figures in private and supplementary healthcare institutions. If these costs are also accounted for, the amount allocated to the treatment of NCDs attributed to the consumption of SSBs would be higher.

The results of this study reveal the impact of the SUS on health and financial costs, which could be lowered if the consumption of SSBs was reduced in the Brazilian population. Notably, the literature presents evidence of successful public health measures implemented in some countries and localities, such as taxation of SSBs, marketing restrictions, mandatory nutrition labeling, and awareness campaigns (2, 40). Specifically, in Brazil, important advancements have been made as a result of efforts by researchers and experts on the subject, such as the new legislation on front nutrition labeling on packaged foods, which provides clearer information to consumers about the main critical ingredients present in ultra-processed foods, such as sugar, fat, and salt (41). However, it is still necessary to break the inertia and political interest that generate actions contrary to health, being urgent and necessary to implement articulated policies to reduce the consumption of SSBs. Therefore, the commitments made by Brazil in the United Nations Decade of Action for Nutrition (2016–2025) and in the Plan of Strategic Actions for the Confrontation of Chronic Diseases and NCDs in Brazil 2021–2030 (42) involve reducing the regular consumption of SSB by at least 30% in the adult population (1).

## Conclusion

This study showed that, in Brazil, in 2019, the consumption of SSBs reflected a significant financial impact on the SUS from medium and high complexity procedures in the treatment of NCDs. These results reveal that if the consumption of SSBs was reduced in Brazil, a relevant financial amount could have been saved in the treatment of these comorbidities; these values could have been reverted to health protection and promotion policies. Despite Brazil’s commitment to reducing the consumption of SSBs, little progress has been made in the policy field in recent years to achieve this goal. There is an urgent need for the implementation and strengthening of articulated actions, especially those based on evidence that considers the Brazilian regional disparities to contribute to the reduction of the high burden that NCDs impose on the population.

## Data availability statement

The original contributions presented in this study are included in the article/supplementary material, further inquiries can be directed to the corresponding author.

## Ethics statement

The studies involving human participants were reviewed and approved by the Comitê de Ética em Pesquisa Envolvendo Humanos–Universidade Federal Ouro Preto. Written informed consent for participation was not required for this study in accordance with the national legislation and the institutional requirements.

## Author contributions

JL conception and study design, analysis and interpretation of data, writing the manuscript, and critical review and final approval. AF and LC analysis and interpretation of data and critical review and final approval. AM conception and study design, analysis and interpretation of data, and critical review and final approval. ÍM conception and study design, project administration, analysis and interpretation of data, critical review, and management of financial resources and final approval. MM conception and study design, analysis and interpretation of data, critical review, supervision, and final approval. All authors contributed to the article and approved the submitted version.
